# A Retrospective Comparison of ACL Tear and Mucoid Degeneration MRI Findings and an Emphasis on Evaluating of ACL, Blumensaat, and PCL Angles

**DOI:** 10.5334/jbsr.1654

**Published:** 2020-07-03

**Authors:** Fatih Celikyay, Ruken Yuksekkaya, Erkal Bilgic

**Affiliations:** 1 Gaziosmanpasa University, TR

**Keywords:** ACL, tear, mucoid degeneration

## Abstract

**Objective:**

To determine MRI findings that can differentiate anterior cruciate ligament (ACL) tears from mucoid degeneration.

**Material and Methods:**

Thirty-seven patients with complete ACL tears and 43 with ACL mucoid degeneration were included in this study. Discontinuity, the abnormal signal intensity of the ACL on fat-saturated-PD weighted images, contusions, a deep lateral femoral notch, anterior tibial translation, uncovered posterior horn of the lateral meniscus, a celery stalk appearance, thickening, ganglion cysts, intraosseous cysts, the ACL, Blumensaat, and posterior cruciate ligament (PCL) angles were evaluated. Optimum threshold values, sensitivity, specificity, and 95% CIs for the angles were calculated to predict the tear.

**Results:**

The prevalence of the significant findings in a tear versus mucoid degeneration, respectively, was as follows: discontinuity (97% vs. 0%, p < 0.001), contusions (65% vs. 2%, p < 0.001), the deep lateral femoral notch (22% vs. 0%, p = 0.001), anterior tibial translation (70% vs. 14%, p < 0.001), uncovered the lateral meniscus (46% vs. 7%, p < 0.001), a celery stalk appearance (0% vs. 66%, p < 0.001), thickening (19% vs. 100%, p < 0.001), ganglion cysts (14% vs. 70%, p < 0.001), and intraosseous cysts (8% vs. 63%, p < 0.001). Threshold values of ACL, Blumensaat, and PCL angles to predict the tear were ≤36° (78% sensitivity, 91% specificity), >11° (84%, 81%), and ≤96° (65%, 91%), respectively.

**Conclusion:**

A celery stalk appearance in the mucoid degeneration and discontinuity in an ACL tear are important in the differential diagnosis. ACL, Blumensaat, and PCL angles can be helpful in settings of diagnostic uncertainty.

## Introduction

Anterior cruciate ligament (ACL) tears are one of the common injuries of the knee and can lead to substantial disability [1]. Multiple primary and secondary signs of an ACL injury on magnetic resonance imaging (MRI) have been described. Primary signs of ACL tears usually include focal or diffuse discontinuity, abnormal signal intensity, and abnormal orientation or bowing of the ACL [1,2,3,4,5,6,7,8,9,10,11,12]. While the abnormal orientation may be visually appreciated, measurement of the ACL and Blumensaat angles can be required to detect subtle changes and improve the accuracy of the MRI [1,3,7,10,11]. Commonly used secondary MRI signs include bone contusions in the lateral femoral condyle and posterolateral tibial plateau, anterior translation of the tibia, uncovered posterior horn of the lateral meniscus, decreased posterior cruciate ligament (PCL) angle, and Segond fracture [1,2,6,7,8,9,10,11,12,13,14,15,16,17]. Although there is no cutoff age for surgery, the treatment of ACL tears in patients younger than 40–50 years of age with high-grade injury is usually reconstruction [18,19].

In contrast to the tear, mucoid degeneration of the ACL characteristically does not cause knee instability [20,21,22]. Recent studies have reported that the prevalence of ACL mucoid degeneration is approximately 2–14% [20,23,24,25]. The main MRI finding of ACL mucoid degeneration is a celery stalk appearance [26,27]. An ill-defined contour, thickening, intraosseous cysts, and ganglia associated with the ACL can also be seen [20,21,22,23,25,26,27]. The treatment is usually based on clinical features [28].

On MRI, increased signal intensity and poor visualization of the ACL do not always correspond to a tear, as mucoid degeneration also has an increased signal on fluid-sensitive images and indistinct margins. It has been suggested that the secondary signs of ACL tear may be helpful in differentiating the tear from ACL mucoid degeneration [21,23]. However, to the best of our knowledge, MRI findings have not been compared between both groups in a large study. Additionally, ACL, Blumensaat, and PCL angles have not yet been tested for differentiation of a torn ACL from mucoid degeneration. Therefore, the purpose of the present study was to elucidate MRI findings that can distinguish between complete ACL tears and mucoid degeneration.

## Material and Methods

### Patient Groups

Local ethics committee approval was obtained for the review of patient records and MRI examinations. We retrospectively reviewed consecutive 3756 knee MRI examinations performed between March 2012 and May 2015 at our institution. Forty-two patients (43 knees) (11 males, 31 females; mean age 54.6 ± 10.7 years; range 30 to 73) (1.15% of 3756 knees MRIs) interpreted as mucoid degeneration of the ACL by the investigator radiologists were included diagnosed in the study. The inclusion criteria for the mucoid degeneration group were the following: available medical record with the relevant history and physical examination; no history of knee surgery and/or known inflammatory arthritis and/or severe osteoarthritis. Patients with ACL instability at the physical examination were not included in the mucoid degeneration group. Thirty-seven patients (34 males, 3 females; mean age 29.4 ± 9.1 years; range 16 to 51), (0.99% of 3756 MRIs) with arthroscopically confirmed complete ACL tears were included in the tear group. The inclusion criterion for the tear group was that the time between the MRI exams and arthroscopy was less than 12 months. We did not include patients with partial ACL tear because the angles will not be so different compared to mucoid degeneration. Data on patient age and gender were recorded. Physical examination, history, and arthroscopy records of the patients were obtained from the hospital electronic medical records.

### MRI Protocol

All imaging studies were performed on a Signa 1.5 T Excite HD MRI system (GE Healthcare, Milwaukee, WI, USA) and a dedicated phased array knee coil with four-channel. The knee was placed in an extended position (0°) while the patient was the supine situation without any rotation at the hip and additional padding to accentuate anterior translation of the tibia. All knee MRI exams included axial, sagittal, and coronal fat-suppressed fast spin-echo proton density (PD) images (echo time [TE]: 30–42, repetition time [TR]: 2100–2800), sagittal T1-weighted images (TE: 9–12, TR: 600–800), and sagittal gradient-echo images (TE: 10–11, TR: 350–400, flip angle: 25°). The number of excitations for sequences was 1–2. A slice thickness of 4 mm with a 0.4–1 mm interslice gap was used. The field of view was 16–18 cm and the matrix size was 384–320 × 320–224.

### Analysis of MR images

Analysis of MR images was performed using the patient archive at our facility and the Centricity RA 1000 picture archiving and communication system (PACS) workstation (GE Healthcare, Milwaukee, WI, USA). All knee MRI examinations were randomly assigned to a list without patients’ history. To avoid any bias, the MRIs were re-assessed by a musculoskeletal radiologist with 13 years of experience one month after completion of the initial assessment. The MRI findings evaluated were the primary and secondary findings of an ACL tear, a celery stalk appearance, ACL thickening, an ill-defined ACL, presence of ganglion cysts associated with the ACL, and intraosseous cysts and bone marrow edema at the femoral and/or tibial attachments.

**Figure 1:**
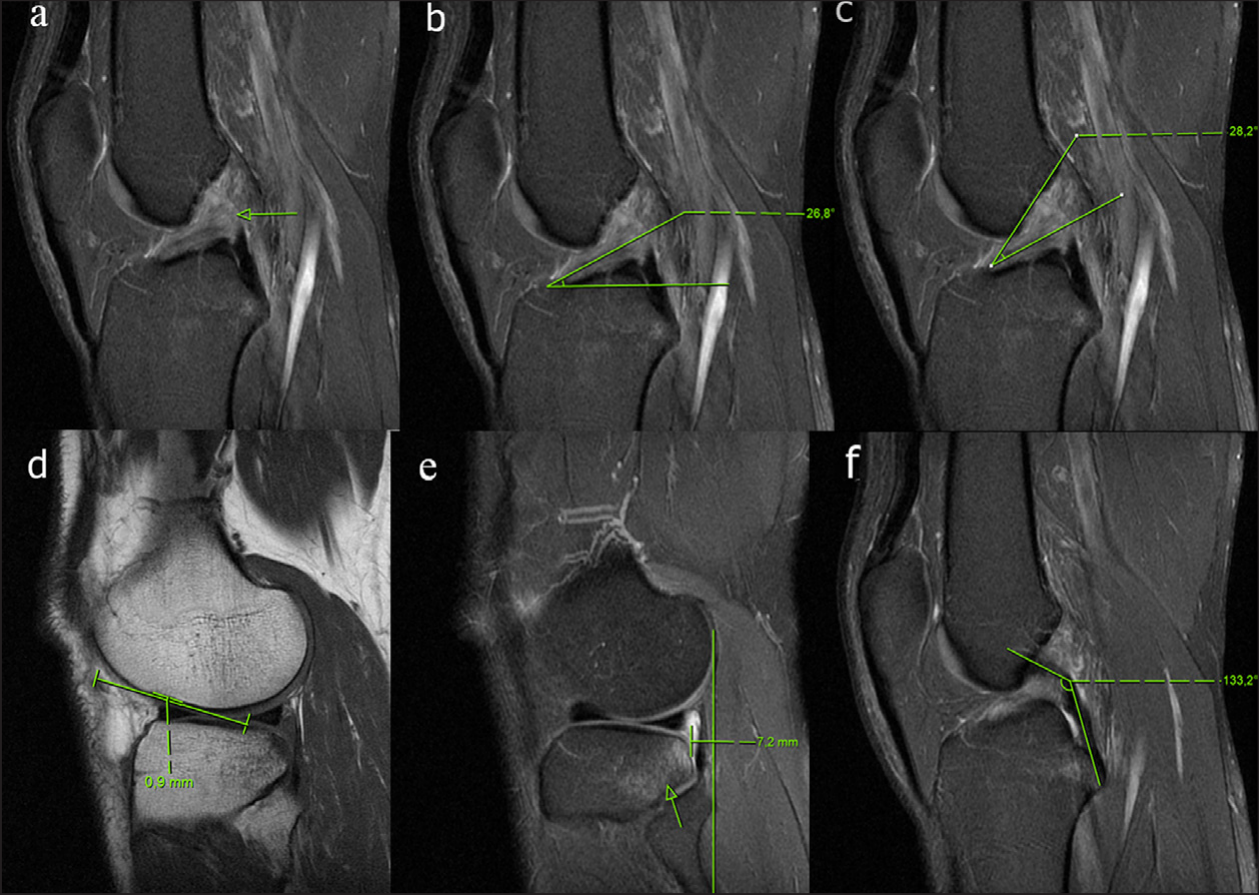
A 33-year-old male patient with a torn ACL, arthroscopically proven. **(a)** Sagittal PD-weighted image demonstrating discontinuity of the ACL (arrow). The ACL angle is decreased **(b)**, consistent with a tear. The apex of Blumensaat angle **(c)** formed distally with a positive value. **(d)** There is no deep the lateral femoral notch sign on the T1 weighted image. **(e)** There is a significant anterior translation of the tibia on the lateral midsagittal PD weighted image. The PCL angle **(f)** is normal.

Primary MRI signs of the tear included focal or diffuse discontinuity, abnormal high-signal intensity on fat-saturated PD-weighted images (diffuse or focal), and an abnormal orientation. Discontinuity (Figure 1a) was defined as a focal gap within the ligament or diffuse disruption of all of its fibers in any imaging plane [11]. The abnormal orientation of the ligament was evaluated by measurement of the ACL (Figure 1b) and Blumensaat angles (Figure 1c). The ACL angle was formed by the intersection of a parallel line to the distal portion of the ACL and a tangential line to the most anterior aspect of the intercondylar eminence which is perpendicular to the long axis of the tibia on a midsagittal MRI image [11]. The Blumensaat angle was formed by the intersection of lines drawn through the distal portion of the ACL and the intercondylar roof. The Blumensaat angles that formed proximally considered negative, while those that formed distally were considered positive [3,7,11].

Secondary MRI signs of the tear included bone contusions in the lateral femoral condyle and/or posterolateral tibial plateau, a deep lateral femoral notch greater than 1.5 mm, an anterior tibial translation greater than 5 mm, uncovered posterior horn of the lateral meniscus, reduction of the PCL angle, Segond fracture, and effusion. The contusion was defined as bone marrow edema more than 5 mm in the anterior femoral condyle and/or posterolateral tibial plateau. The deep lateral femoral notch (Figure 1d) was measured on the lateral femoral condyle at the adjunction of the weight-bearing tibial and patellar articular surfaces of the femoral condyle [13]. Anterior tibial translation (Figure 1e) was quantified by measuring the distance between two parallel lines drawn a tangent to the posterior of the lateral femoral condyle and the tibial plateau on the midsagittal image through the lateral compartment of the knee [1,6]. The anterior position of the tibia relative to the femoral condyle had a positive value. An uncovered posterior horn of the lateral meniscus was defined as the posterior displacement of the lateral meniscus relative to the posterior vertical margin of the mid-lateral tibial plateau [7,16,17]. The PCL angle (Figure 1f) was the angle formed between the lines tangential to the proximal and distal parts of the PCL [7,11,17].

**Figure 2:**
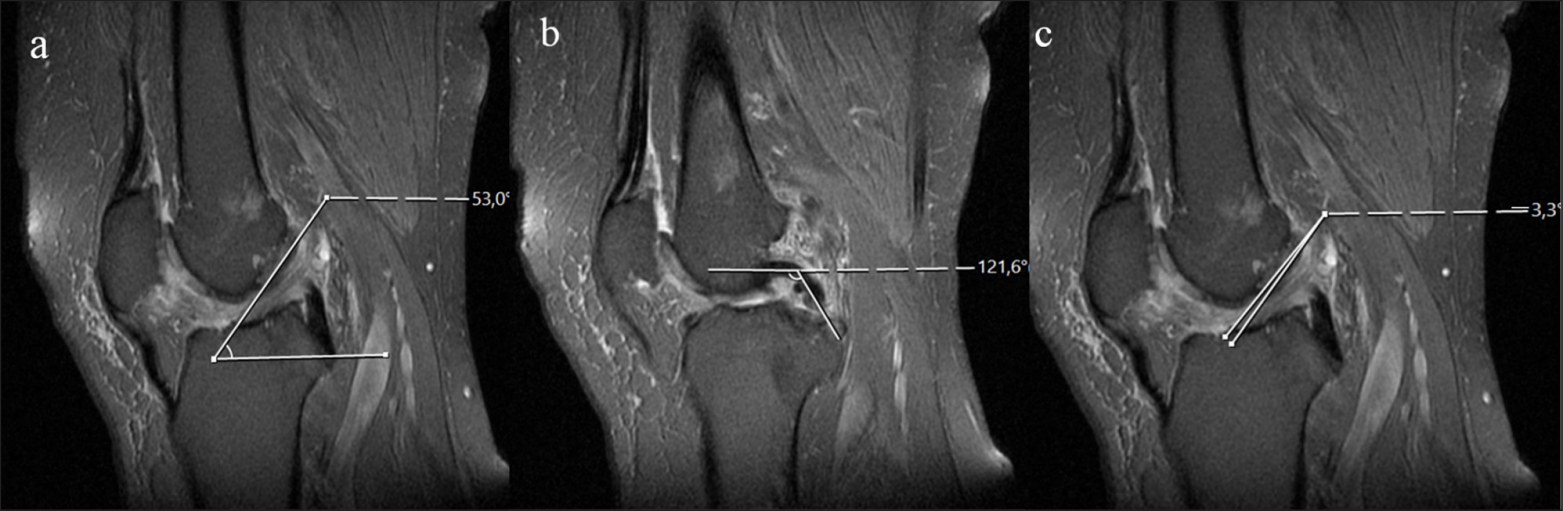
A 50-year-old female patient with mucoid degeneration. Sagittal PD-weighted images show the ACL **(a)** and PCL angles **(b)** are in the normal range. The ACL (a) also has a celery stalk appearance. The apex of Blumensaat angle **(c)** formed proximally with a negative value, which does not indicate a tear.

**Figure 3:**
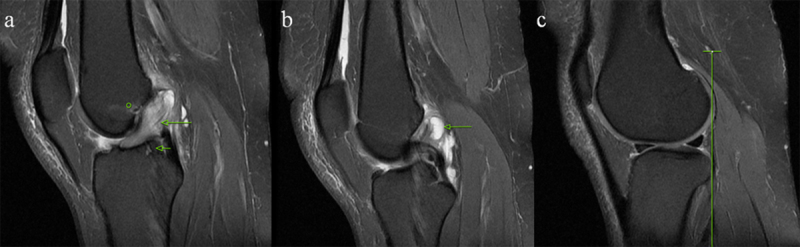
A 47-year-old female patient with mucoid degeneration. **(a)** Sagittal PD-weighted image demonstrating increased signal and poor visualization of the thickened ligament, consistent with a celery stalk appearance (long arrow), bone marrow edema at the femoral origin (circle), and an intraosseous cyst at the tibia insertion (short arrow). Consecutive images show a ganglion cyst (arrow) **(b)** associated with the ACL and lack of anterior tibial translation **(c)**.

A celery stalk (Figures 2a and 3a) sign was described the appearance of intact fibers with low signal separated from each other by a prominent high signal within thickened ACL on fat-saturated PD-weighted images [26,27]. The ACL thickening was defined when the thickness was not uniform and 7 mm or greater [11]. The criterion for ill-defined ACL was contour of the ligament poorly seen on T1-weighted and fat-saturated PD-weighted images. Ganglion cysts associated with the ACL were defined as fluid signal adjacent to or within the ligament having the following criteria: mass effect on anterior cruciate ligament fibers and lobulated margin (Figure 3b) [20].

### Data analysis

All MRI findings were compared between the groups. Statistical analysis was performed using SPSS version 18.0 for Windows software package (SPSS Inc., Chicago, IL, USA). A nonparametric Wilcoxon 2-sample *t*-test was used to compare continuous variables, while categorical variables were compared using a chi-square test. Continuous data were expressed as means ± standard deviations, and categorical data were expressed as numbers (*n*) with related percentages. Statistical significance was set at an alpha of 0.05. The most appropriate threshold values for the ACL, Blumensaat, and PCL angles were calculated with univariate logistic regression analysis. The sensitivity and specificity of the angles were determined and obtained receiver operating characteristic (ROC) curves. Odds ratios and 95% confidence intervals (CI) were calculated using logistic regression analysis.

## Results

**Figure 4:**
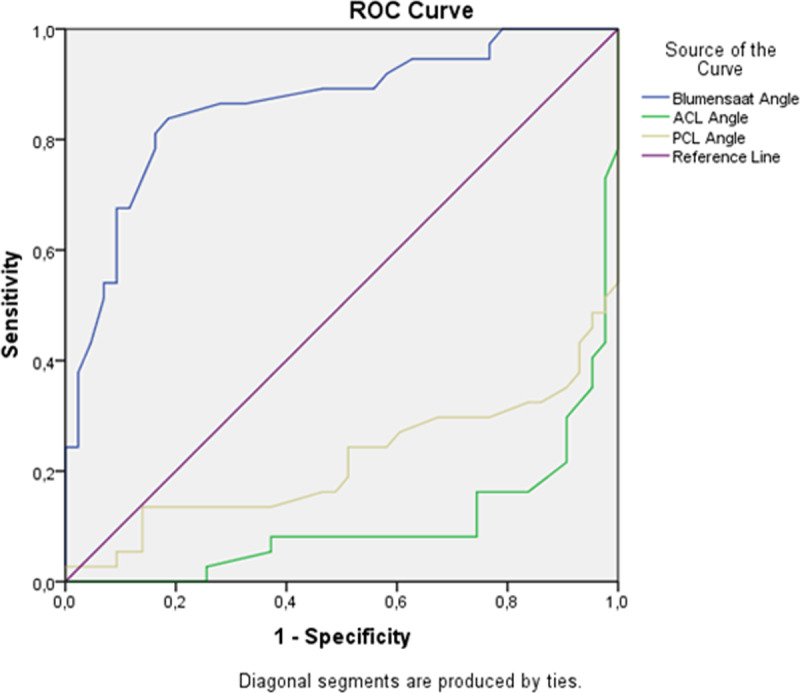
ROC curves for ACL, Blumensaat, and PCL angles. Areas under the curves (AUC) were 0.894 for the ACL angle, 0.861 for the Blumensaat angle, and 0.784 for the PCL angle.

Tables 1, 2 list results of all MRI findings evaluated. The following is a summary of the MRI findings. Discontinuity of the ACL was observed in almost all tear cases but was absent in the mucoid degeneration group (97% vs.0%, respectively; *P* < 0.001). The bone contusions were significantly more frequently observed with an ACL tear, but rare with mucoid degeneration (65% vs. 2%, respectively; *P* < 0.001). An anterior tibial translation greater than 5 mm was more common in the ACL tear group than the mucoid degeneration group (70% vs.14%, respectively; *P* < 0.001). A celery stalk appearance was only seen in the mucoid degeneration group (66% vs. 0%; *P* < 0.001). Ganglion cysts associated with the ACL (70% vs. 14%; *P* < 0.001) and intraosseous cysts at the sites of the ligament attachment to the bones (63% vs. 8%; *P* < 0.001) were significantly more common in the mucoid degeneration versus the tear group, respectively. ACL thickening was found in all knees with mucoid degeneration, however was uncommon in patients with the tear (100% vs.14%; *P* < 0.001) (Table 1). The mean values, optimum thresholds, and ROC analysis results of the angles can be found in Table 2 and Figure 4.

**Table 1:** The MR findings in ACL tear and mucoid degeneration groups.

	ACL tear group (n = 37) (%)	ACL mucoid degeneration group (n = 43) (%)	*P* value

**Discontinuity**	36 (97)	0 (0)	<0.001
**ACL with high signal on fat-sat PDW images**	37 (100)	43 (100)	—
**The lateral bone contusions**	24 (65)	1 (2)	<0.001
**>1.5 mm of deep lateral femoral notch**	8 (22)	0 (0)	0.001
**>5 mm of anterior tibial translation**	26 (70)	6 (14)	<0.001
**Uncovered meniscus sign**	17 (46)	3 (7)	<0.001
**Effusion**	25 (68)	23 (54)	0.292
**Segond fracture**	0 (0)	0 (0)	—
**Celery stalk appearance**	0 (0)	28 (66)	<0.001
**Thickening**	7 (19)	43 (100)	<0.001
**Ill-defined ACL**	20 (54)	43(100)	<0.001
**Ganglion cyst associated with the ACL**	5 (14)	30 (70)	<0.001
**Intraosseous cyst**	3 (8)	27 (63)	<0.001
**Bone marrow edema at the ACL attachment**	6 (17)	10 (23)	0.657

**Table 2:** Results of the ACL, Blumensaat, and PCL angles.

	ACL mucoid degeneration (mean ± SD)	ACL tear (mean ± SD)	The optimum Cutoff*	Sensitivity*% (95% CI)	Specificity*% (95% CI)

**ACL angle**	45.1° ± 7.9°	32° ± 7.1°	≤36°	78.3 (61.8–90.2)	90.7 (77.9–97.4)
**Blummensaat angle**	6.4° ± 8.5°	19.8° ± 9.4°	>11°	83.8 (68–93.8)	81.4 (66.6–91.6)
**PCL angle**	111.9° ± 12.5°	92.3° ± 21°	≤96°	64.8 (47.5–79.8)	90,7 (77.9–97.4)

* To predict torn ACL.

## Discussion

In the present study, discontinuity of the ACL was a useful finding for differentiating a tear from mucoid degeneration. The high signal intensity on fat-suppressed PD-weighted images was seen in all tear and mucoid degeneration cases in our study. ACL and Blumensaat angles are objective tests for evaluating the horizontal displacement of the distal ligament. It has been reported the mean ACL and Blumensaat angles in patients with a torn ACL were 23.9°–33.9° and 21.4°–25.8°, respectively (Table 3) [3,7,10,11]. In our study, the mean ACL and Blumensaat angles in the tear group were, respectively, 32° ± 7.1° and 19.8° ± 9.4°. These results were consistent with the previous studies. However, we observed that the mean ACL angle (45.1° ± 7.9°) in the mucoid degeneration group was lower than that (52.3°–55.6°) in individuals with normal ACLs when compared with the previous studies (Table 3). Additionally, we found the mean Blumensaat angle in the mucoid degeneration group was 6.4° ± 8.5°, although this angle has been reported to have slightly negative values in healthy individuals (Table 3). These results may be secondary to changes in the orientation due to mucoid content within the ACL with mucoid degeneration.

**Table 3:** Review of the literature for ACL, Blumensaat, and PCL angles.

	Gentili et al. (3)*	Lee et al. (7)*	Murao et al. (10)*	Mellado et al. (11)*	The current studyα

**Position of the knee on MRI examination**	Full extension	Full extension	Full extention or slight flexion	—	Full extension
**ACL angle**					
Intact ACL	55.6°	—	52.3°	53.5°	45.1°
Torn ACL	29.9°	—	33.9°	25.9°	32°
Cutoff	<45°	—	≤45°	≤45°	≤36°
Sensitivity% (95% CI)	91	—	93	100 (92–100)	78 (62–90)
Specificity% (95% CI)	97	—	84	100 (92–100)	91 (78–97)
**Blummensaat angle**					
Intact ACL	–1.6°	–4.1°	—	–8.2°	6.4°
Torn ACL	25.8°	27.9°	—	21.4°	19.8°
Cutoff	>9°	>9°	—	>0°	>11°
Sensitivity% (95% CI)	91	94 (70–100)	—	90 (78–96)	84 (68–94)
Specificity% (95% CI)	86	96 (75–100)	—	98 (89–99)	81 (67–92)
**PCL angle**					
Intact ACL	123°	122°	—	128.9°	111.9°
Torn ACL	106°	105.7°	—	111.5°	92.3°
Cutoff	<107°	<114°	—	<115°	≤96°
Sensitivity% (95% CI)	52	74 (51–96)	—	70 (55–82)	65 (48–80)
Specificity% (95% CI)	94	71 (51–91)	—	82 (68–91)	91 (78–97)

* The studies about the distinction of ACL tear from intact ACL. Intact ACL refers to normal ACL. ^α^ The current study. Intact ACL refers to mucoid degeneration of the ACL. — Not calculated or not available.

The previous studies have also indicated that an ACL angle less than 45° and Blumensaat angle greater than 0–9° were strongly associated with a tear (Table 3) [3,7,10,11]. In the current study, there was some overlap in the reported threshold angles for the groups. We found that an ACL threshold angle of 36° or less produced 78.3% sensitivity (95% CI: 61.8–90.2%) and 90.7% specificity (95% CI: 77.9–97.4%). Moreover, using a Blumensaat angle greater than 11° resulted in a sensitivity of 83.8% (95% CI: 68.0–93.8%) and 81.4% specificity (95% CI: 66.6–91.6%) in the differentiation of tears from mucoid degeneration. They were useful for the differentiation in our study. However, the sensitivity and specificity observed for the ACL and Blumensaat angles were usually some lower than that reported for differentiating torn from normal ACLs (Table 3).

In the literature, the highest specificities of the secondary findings for ACL tears have been noted as a deep lateral femoral notch, bone contusions, an anterior tibial translation of 5 mm or more, and uncovering of the posterior horn of the lateral meniscus [2,11,15,16,17]. In our study, these signs were found to be useful for distinguishing tears from mucoid degeneration. The PCL angle has been reported to be decreased in knees with a torn ACL [3,7,11,17]. Results from those studies also indicate that using a threshold value of 105–115° for the PCL angle may be useful as a prediction of an ACL tear. The present study determined that the optimum threshold PCL angle for differentiating ACL tears from mucoid degeneration was 96° or less with 64.8% sensitivity (95% CI: 47.5–79.8%) and 90.7% specificity (95% CI: 77.9–97.4%) (Tables 2 and 3). However, the diagnostic performance of the PCL angle measurement was lower than that of the ACL angle.

The present study showed that a celery stalk appearance, ill-defined ACL, thickening, the ganglion cysts, and intraosseous cysts could be reliable MRI findings for distinguishing the mucoid degeneration from the tear. This outcome was coherent with previous studies involving only cases of the mucoid degeneration [20,21,22,23]. On the other hand, some studies have reported that diffuse thickening of the ACL is also an MRI finding suggestive of the tear [4,5]. Herein, ACL thickening was rare in patients with the tear and more frequent with mucoid degeneration (14% vs. 70%, respectively).

Our study had some limitations. First, this study was retrospective. Also, ACL mucoid degeneration diagnoses were not confirmed arthroscopically or histologically. However, patients who had any suspicion of the ACL insufficiency at the physical examination in the hospital electronic medical records not included in the degeneration group. Another limitation is the knee joint position in the MRI examination, which can affect the angle measurements, as mentioned by Guenoun et al. [29]. However, we routinely obtained all knee MRI examinations during full extension of the knee similar to most of the studies compared [3,7,10], but the position of the knee in the study by performed Mellado et al. [11] was not exactly stated. Finally, we did not evaluate intra- and interobserver variability.

## Conclusion

In summary, the anterior tibial translation, uncovering of the lateral meniscus, and particularly the lateral bone contusions, a deep femoral notch can suggest a complete ACL tear, while ACL thickening, ill-defined ACL, ganglion cysts and intraosseous cysts at the ligament attachments can be helpful for the diagnosis of ACL mucoid degeneration. More importantly, radiologists should be familiar with the celery stalk appearance in mucoid degeneration of ACL and discontinuity in a torn ACL for differentiation. Measurement of the ACL angle, as well as Blumensaat and PCL angles, can be helpful in settings of diagnostic uncertainty.
